# All That Glitters is not Gold! A Case of Concomitant Acute Pericarditis and Subsegmental Pulmonary Embolism

**DOI:** 10.31138/mjr.32.1.88

**Published:** 2021-02-08

**Authors:** Dimitrios Patoulias, Savvas Papachristou, Dimitrios Vitos, Xanthi Apostolidou, Vivian Georgopoulou, Andreanna Kozanidou, Dafni Stamou, Nikolaos Samarentsis, Andriana Chioni, Spyridon Bakatselos

**Affiliations:** 1 First Department of Internal Medicine, General Hospital “Hippokration”, Thessaloniki, Greece; 2 Second Department of Cardiology, Aristotle University of Thessaloniki, Thessaloniki, Greece; 3 Department of Radiology, General Hospital “Hippokration”, Thessaloniki, Greece

**Keywords:** Acute pericarditis, pulmonary embolism, pericardial effusion, systemic lupus erythematosus

## Abstract

Concomitance of acute pericarditis and pulmonary embolism is extremely rare, with only a few case reports published so far. Herein we present a case of a 50-year-old man that presented to the Emergency Department, complaining of fever up to 38.5°C, pleuritic chest pain, nausea, arthralgias, and general symptoms during the previous two weeks. Thorough diagnostic work-up revealed the diagnosis of concomitant acute pericarditis and pulmonary embolism, which raised high index of clinical suspicion for systemic lupus erythematosus (SLE). Indeed, the patient did not marginally meet the diagnostic criteria for SLE (total score=8), according to the updated 2019 European League Against Rheumatism/American College of Rheumatology Classification Criteria. Since then, the patient remains asymptomatic, while he is under close monitoring for potential manifestation of other SLE clinical features. Our case highlights the need for long-term follow-up in such patients, especially when the first episode is attributed as idiopathic.

## CASE DESCRIPTION

A 50-year-old man presented to the Emergency Department on March 2020, complaining of fever up to 38.5^°^C, pleuritic chest pain, nausea, arthralgias, and general symptoms during the previous two weeks, after returning from an occupational travel. His past medical history was unremarkable, except for having a first degree relative diagnosed with systemic lupus erythematosus (SLE), while the patient did not report smoking, alcohol, or drug abuse. Physical examination did not reveal any pathological signs, except for diminished breath sounds in the left lung base. Patient was febrile on admission, while the rest of his vital signs were normal. Electrocardiogram revealed sinus rhythm, while arterial blood gas examination was normal. A mild left pleural effusion was depicted in chest X-ray. Initial laboratory exams revealed leucocytosis (15300/mm^3^) with polynucleosis (82%), mild thrombocytosis (490000/μL), elevated C-reactive protein (242 mg/L, normal value: <6 mg/L) and fibrinogen levels (724 mg/dL, normal range: 150–380 mg/dL), elevated D-dimer levels (9853 μg/L, normal range: 0–500 μg/L), and normal renal function. Calculated Well’s score was zero. Therefore, the patient was admitted for further investigation.

**Figure 1.**
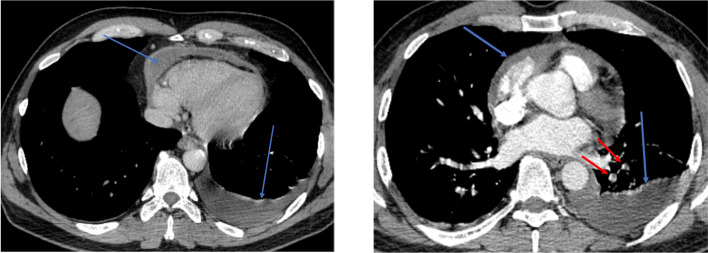
Chest CT scan: (A) notice the pericardial effusion, the left pleural effusion and (B) the bilateral, subsegmental pulmonary embolism (arrows).

Blood cultures were negative, while further laboratory testing demonstrated normal procalcitonin levels, high ferritin levels (2128 ng/mL, normal range: 10–291 ng/ mL), and elevated erythrocyte sedimentation rate (90 mm/h). Patient was placed on chemoprophylaxis with broad spectrum antibiotics (b-lactam plus macrolide) and low-molecular weight heparin. Testing for influenza A and B, hepatitis B virus (HBV), hepatitis C virus (HCV), human immunodeficiency virus (HIV), severe acute respiratory syndrome coronavirus-2 (SARS-CoV-2), Legionella pneumophila, Streptococcus pneumoniae, and Mycobacterium species was also negative. Due to persistent fever without signs of recession, patient underwent a chest and abdomen computed tomography (CT) scan, during the second day of hospitalisation. The latter revealed the presence of both acute pericarditis and distal, subsegmental pulmonary embolism, along with bilateral pleural effusion (**Figure 1A,B**). An echocardiogram was performed, confirming the presence of moderate pericardial effusion (14 mm), without signs of right ventricular dysfunction. The patient then received ibuprofen and colchicine, along with low-molecular weight heparin at therapeutic dose. Two days later he was afebrile. Repeated echocardiogram revealed a decrease in pericardial effusion (14 χ 9 mm). Doppler ultrasound exam of legs ruled out the presence of deep vein thrombosis.

The diagnosis of acute pericarditis, based on the respective 2015 European Society of Cardiology Guidelines (two criteria were fulfilled, namely pleuritic pain and new pericardial effusion, while, no pericardial friction-rub or electrocardiogram changes were present), led to additional laboratory exams, which ruled out several major causes of acute pericarditis, such as infectious, neoplastic, metabolic or drug-related.^1^ However, the concomitance of acute pericarditis and pulmonary embolism raised high clinical suspicion of underlying SLE with or without secondary antiphospholipid syndrome (sAPS). Indeed, thorough immunologic investigation revealed a positive titre of anti-nuclear antibodies (ANA) equal to 1:160 and marginally positive lupus anticoagulant. The rest immunologic profile (anti-dsDNA, anti-ENA, anti-Sm, anti-CCP, c-ANCA, p-ANCA, anti-cardiolipin and anti-β2-glycoprotein antibodies, C3, C4) along with testing for inherited thrombophilia were negative. Repeating measurement of antiphospholipid antibodies 12 weeks later however revealed that all were negative.^2^ Thus, the patient did not fulfil the diagnostic criteria for SLE (total score=8), according to the updated 2019 European League Against Rheumatism/American College of Rheumatology Classification Criteria.^3^

He was discharged home one-week post-admission, in excellent general condition, with recommendation to continue ibuprofen and colchicine treatment, while he was also prescribed low-molecular weight heparin at therapeutic dose initially, which was substituted with direct oral anticoagulant after negative repeating evaluation of antiphospholipid antibodies. Two months later, patient remains asymptomatic, while he is under close monitoring for potential manifestation of other SLE clinical features.

## CONCLUSION

After a comprehensive research of the relevant literature, we found only a few case reports describing the coexistence of acute pericarditis and pulmonary embolism.^4,5^ Autoimmune diseases should always be ruled out, despite being a relatively uncommon cause of acute pericarditis.^1^ Dressler syndrome after acute pulmonary embolism should also be considered,^6,7^ despite the decreased incidence rates of the syndrome.^8,9^ Pericarditis is relatively common among SLE patients, even if it does not necessarily associate with clinical symptoms.^10,11^ Patients require long-term follow-up for potential recurrence or other clinical manifestations, especially when the first episode is attributed as idiopathic.
